# Correction: Prevalence of major depressive disorder and its determinants among young married women and unmarried girls: Findings from the second round of UDAYA survey

**DOI:** 10.1371/journal.pone.0309220

**Published:** 2024-08-15

**Authors:** Shromona Dhara, Joyeeta Thakur, Neelanjana Pandey, Arupendra Mozumdar, Subho Roy

The funding statement for this paper is incorrect. The correct funding statement is: “Data collection for the UDAYA survey was supported by a grant to the Population Council from the Bill & Melinda Gates Foundation (grant OPP 1111281) and the David and Lucile Packard Foundation (2014–40467). The funders had no role in the data analysis, interpretation, writing, or submission of this article.”

The following information is missing from the Acknowledgments section: “We are grateful to the Bill & Melinda Gates Foundation (Grant numbers: INV-009814, OPP1111281) for their financial support towards the publication of this research in PLOS ONE.”

There is an error in affiliation 2 for author Joyeeta Thakur. The correct affiliation 2 is: DABS-I, Indian Council of Medical Research, Hyderabad, India.

The ORCID iDs are missing for the second, third, fourth, and fifth author. Please see the authors’ respective ORCID iDs here:

Author Joyeeta Thakur’s ORCID iD is: 0000-0001-5699-6052 (https://orcid.org/0000-0001-5699-6052).

Author Neelanjana Pandey’s ORCID iD is: 0000-0003-3018-099X (https://orcid.org/0000-0003-3018-099X).

Author Arupendra Mozumdar’s ORCID iD is 0000-0002-2170-2656 (https://orcid.org/0000-0002-2170-2656).

Author Subho Roy’s ORCID iD is 0000-0002-6648-4875 (https://orcid.org/0000-0002-6648-4875).
