# Supplementary material for: Prevalence of major depressive disorder and its determinants among young married women and unmarried girls: Findings from the second round of UDAYA survey
Source: PLoS One. 2024 Jul 2;19(7):e0306071. doi: 10.1371/journal.pone.0306071 (PMC11218953; doi:10.1371/journal.pone.0306071)
Supplement: S1 Table — (DOCX) [file pone.0306071.s001.docx]

S 1 Variables used to compute Level of depression using the PHQ-9 scoring method.

| **Variable description** | **Coding** | **Category** |
| --- | --- | --- |
| Had trouble falling asleep, or sleeping too much in the last 2 weeks | Coded **not at all = 0, less than one week = 1, One-week or more =2 and nearly every day = 3.** Added the numbers to make the PHQ score. (Out of 27) | Ref. No/mild depression (PHQ sc. 0-9)  Moderate to severe depression (PHQ sc. 10-27) |
| Been feeling tired or having little energy in the last 2 weeks |  |  |
| Had been poor appetite or overeating in the last 2 weeks |  |  |
| Had trouble concentrating on things in the last 2 weeks |  |  |
| Had little interest or pleasure in doing things in the last 2 weeks |  |  |
| Been feeling down, depressed or hopeless in the last 2 weeks |  |  |
| Been feeling bad about yourself in the last 2 weeks |  |  |
| Have been moving or speaking so slowly in the last 2 weeks |  |  |
| Had thoughts that you would be better off dead in the last 2 weeks |  |  |
