# Supplementary material for: Prevalence of major depressive disorder and its determinants among young married women and unmarried girls: Findings from the second round of UDAYA survey
Source: PLoS One. 2024 Jul 2;19(7):e0306071. doi: 10.1371/journal.pone.0306071 (PMC11218953; doi:10.1371/journal.pone.0306071)
Supplement: S2 Table — (DOCX) [file pone.0306071.s002.docx]

S 2 List of variables and assessment of Composite score of Self-efficacy

| **Variable description** | **Categories and coding** |
| --- | --- |
| Respondent chooses how to spend free time | Completely sure=5, Somewhat sure=4, Neither sure-unsure=3, Somewhat unsure=2 Not at all sure=1 |
| Respondent participates in no-family/non-school related events/functions |  |
| Respondent chooses to earn an income if he/she wishes to |  |
| Respondent talks freely to her parents/in-laws about their aspirations |  |
| Respondent expresses choice in the type of clothing for self |  |
| Respondent plays a strong role in resolving family conflicts |  |
