# Supplementary material for: Prevalence of major depressive disorder and its determinants among young married women and unmarried girls: Findings from the second round of UDAYA survey
Source: PLoS One. 2024 Jul 2;19(7):e0306071. doi: 10.1371/journal.pone.0306071 (PMC11218953; doi:10.1371/journal.pone.0306071)
Supplement: S3 Table — (DOCX) [file pone.0306071.s003.docx]

S 3. List of variables, assessments, and categories used to compute IPV against the young married women

| **Variable description** | **Coding** | **Category** |
| --- | --- | --- |
| Last 12 months. Respondent’s husband humiliates in front of others | Yes=1, No=0. Added the obtained numbers to assess composite score. (median=1) | Experienced more violence ≥2, Experienced less violence<2. |
| Respondent’s husband ever slapped in the last 12 months |  |  |
| In the last 12 months, twisted arm or pulled hair |  |  |
| In the last 12 months, thrown something |  |  |
| In the last 12 months, something that could hurt |  |  |
| In the last 12 months, dragged or beaten up |  |  |
| In the last 12 months, choked or burnt on purpose |  |  |
| In the last 12 months threaten or attack with any other weapon |  |  |
