# Supplementary material for: Prevalence of major depressive disorder and its determinants among young married women and unmarried girls: Findings from the second round of UDAYA survey
Source: PLoS One. 2024 Jul 2;19(7):e0306071. doi: 10.1371/journal.pone.0306071 (PMC11218953; doi:10.1371/journal.pone.0306071)
Supplement: S4 Table — (DOCX) [file pone.0306071.s004.docx]

S 4 Variables and assessments for evaluating men's opinion about women in work and family life.

| **Item description** | **Coding** | **Categories** |
| --- | --- | --- |
| Boys do as much domestic work as girls | Yes=1, No=0. Added the obtained numbers to assess composite score. | More gendered opinion= scored 3 to 8, and less gendered opinion= scored ≤2. |
| Wrong for a girl to have male friends |  |  |
| Girls like to be teased by boys |  |  |
| Girls are allowed to decide when they want to marry |  |  |
| Father/Husband alone/mainly decide how household money is to be spent |  |  |
| Better for girls to get married early than to complete at least class 12 |  |  |
| Giving the kids a bath and feeding the kids women’s responsibilities only |  |  |
| There are times when a wife deserves to be beaten by her husband |  |  |
