# Supplementary material for: Prevalence of major depressive disorder and its determinants among young married women and unmarried girls: Findings from the second round of UDAYA survey
Source: PLoS One. 2024 Jul 2;19(7):e0306071. doi: 10.1371/journal.pone.0306071 (PMC11218953; doi:10.1371/journal.pone.0306071)
Supplement: S5 Table — (DOCX) [file pone.0306071.s005.docx]

S 5 Variables that were used to assess perceived sibling inequality by the respondents

| **Item description** | **Categories** | **Coding** |
| --- | --- | --- |
| Given fewer amounts of food/ lesser quality food | Inequality is perceived (If answered yes in any item); Inequality is not perceived (Otherwise) | Yes (1), No (0) |
| Given less pocket money |  | Yes (1), No (0) |
| Made to study less/ in a poorer quality school |  | Yes (1), No (0) |
| Given less freedom |  | Yes (1), No (0) |
| Treat with less favor in any other way |  | Yes (1), No (0) |
