# Supplementary material for: Prevalence of major depressive disorder and its determinants among young married women and unmarried girls: Findings from the second round of UDAYA survey
Source: PLoS One. 2024 Jul 2;19(7):e0306071. doi: 10.1371/journal.pone.0306071 (PMC11218953; doi:10.1371/journal.pone.0306071)
Supplement: S1 File — (DOCX) [file pone.0306071.s006.docx]

S1 1. List of independent variables (Individual level)

| **Description** | **Categories and coding** |
| --- | --- |
| **Variables for young married women and unmarried girls** |  |
| *Socio-economic factors* |  |
| Age in completed years | 12-17; 18-23 |
| Social group | Scheduled caste or scheduled tribe; other backward classes (OBC), general |
| Religion | Hindu, Non-Hindu (Muslims, Christians, Sikh, Buddhist or Neo Buddhist, Jain, and atheist) |
| Completed years of schooling | no schooling; Standard 1 to 8; Standard 9 to 10; Standard 11+ |
| State specific wealth index | Low; middle; high (using quartile distribution of wealth score) |
| Age in completed years | 12-17; 18-23 |
|  |  |
| *Working status* |  |
| Done any paid work in last one year | Yes, no |
| Currently looking for a job | Yes, no |
|  |  |
| *Social Capital* |  |
| Currently attending school or any educational course | Yes, no |
| Number of friends | Categorized using median: 0 to 3; 4 or more |
| Been a member of adolescent/youth groups in last three years (KSY/ SABLA, NYKS, SHG, sport) | Yes, no |
|  |  |
| *Agency*  *Participation in decision making* |  |
| Took decision on how money earned the last year should be used | Yes; Did not decide herself or did not earn any money |
| Took decision on how much education should have/till which standard | Yes, no |
| Took decision about who would be friends | Yes, no |
| Took decision about making major household purchases | Yes, no |
| Took decision about whether to work or stay at home | Yes, no |
| Took decision about seeking treatment if feeling sick | Yes, no |
| Self-efficacy: Self-efficacy scores | Responses to six questions in five-point Likert scale was used to compute the score. Categories are: 5-15; 6-20; 21-24 |
|  |  |
| *Experience of harassment* |  |
| Experienced telephonic or online harassment (in last three years) | Yes, no |
|  |  |
| *Physical activity* |  |
| Duration of (in hours) of work, play or exercise hard enough to sweat and breathe heavily (in last week) | No or less than 1 hour of work; More than one hour |
| Duration of (in hours) of screentime: watching television/ video or playing video/ mobile games (in last week) | 0 to 2 hours; 3 to 15 hours; more than 15 hours |
|  |  |
| **Variables for unmarried girls (only)** |  |
| *Interaction with parents in last year* |  |
| Discussed about school performance | Yes, no |
| Discussed about friendship | Yes, no |
| Discussed about being teased by mother or father | Yes, no |
| Discussed about menstruation | Yes, no |
| Discussed about pregnancy | Yes, no |
|  |  |
| *Physical abuse by parents* |  |
| Physically hurt by father or mother of both in the last one year | Yes, no |
|  |  |
| **Variables for young married women (only)** |  |
| *Reproductive history* |  |
| Age at marriage (in years) | 7 to 14; 15-17; 18-19; 20-22 |
| Currently living with husband | Couple lives together; Couple stays apart |
| Reported last sexual intercourse | Days ago; Weeks ago; Months ago; Years ago |
| Ever given birth to a live child | Yes, no |
| Had experienced miscarriage/stillbirth | Yes, no |
| Had experienced induced abortion |  |
| Ever given birth to a live child | Yes, no |
| In-laws pressurize to conceive immediately after marriage or would call barren | Yes, no |

S1 2. List of independent variables (Community level)

| **Description** | **Categories and coding** |
| --- | --- |
| **Estimates that were calculated using data from both young married women and unmarried girls** |  |
| *Sexual assaults on women* |  |
| Experienced eve teasing, molestation, attempted rape, or rape in last three years | Reported by less than 25% respondents;  Reported by 25–50% of respondents;  Reported by more than 50% of respondents |
| **Estimates that were calculated using data only from married girls** |  |
| Experienced dowry-related humiliation | Reported by less than 25% respondents  Reported by 25–35% of respondents  Reported by more than 35% of respondents |
| Reported inter-partner violence (IPV) in the last one year | Reported by less than 15% respondents  Reported by 15-25% of respondents  Reported by more than 25% of respondents |
| **Estimates that were calculated using data only from unmarried girls (Variables used for unmarried girls only)** |  |
| Reporting of family violence: father beaten mother | Does not have parents or reported no violence  Reported by less than 10% of respondents  Reported by 10% or more respondents |
| Reporting of any kind of sibling inequality | Reported by less than 0-15% respondents  Reported by 16–30% of respondents  Reported by more than 30% of respondents |
| **Estimates that were calculated using data only from unmarried boys** |  |
| Boys with gendered attitude towards women | Reported by less than 25% of respondents  Reported by 25 to 45% of respondents  Reported by more than 45% of respondents |
