# Supplementary material for: Prevalence of major depressive disorder and its determinants among young married women and unmarried girls: Findings from the second round of UDAYA survey
Source: PLoS One. 2024 Jul 2;19(7):e0306071. doi: 10.1371/journal.pone.0306071 (PMC11218953; doi:10.1371/journal.pone.0306071)
Supplement: Attachment — Submitted filename: Response to Reviewers.docx [file pone.0306071.s007.docx]

To

The Academic Editor,

PLOS ONE

We express our sincere gratitude to the reviewers for their insightful comments. We have closely gone through all the comments and revised the manuscript accordingly. In the following table we have stated the responses to each point raised by the reviewers.

| **Reviewer #1:** | **Comments** | **Responses** |
| --- | --- | --- |
| 1. | The title of the article includes the phrase ‘major depressive symptoms’ but I do not find it defined anywhere in the text. This phrase is quite confusing as it matches only partially with the standard International Classification of Disease (ICD) terminology. ICD uses the phrase major depressive disorders. Since authors have used PHQ-9, I would suggest them to stick with the word ‘depression’ or the phrase ‘major depressive disorder’, but not ‘major depressive symptom’. | *We agree that aligning our terminology with standard ICD classifications is important. We have revised the manuscript to use the phrase 'major depressive disorder' instead of 'major depressive symptoms' through out to ensure clarity and consistency.* |
| 2. | Abstract’s methodology section does not contain any information on what your dependent variable is and how it has been defined or created. | *The description of dependent variable was incorporated in the methodology section of the abstract.* |
| 3. | Abstract: Please include the age group these women belong to. Also, the year UDAYA data is from. | *The age ranges 12-23 years for both the group of girls are mentioned in this abstract.* |
| 4. | Abstract: ‘major depression’ does not make sense. Please choose one word or phrase, define it properly, and use it  throughout the text to avoid confusing the reader. | *the phrase 'major depressive disorder' (MDD) is used in this revised manuscript, through out.* |
| 5. | Please include only major findings and do provide statistical details, for example, odds ratio and their p-values or confidence intervals, in the abstract. | *The authors have incorporated some of the important findings in the abstract along with the statistical details in thus is revision.* |
| 6. | Targeted interventions – who needs to be targeted as per your findings? You may restructure the conclusive sentences to make it clear | *Authors have modified the conclusive sentences in the abstract. The revised statements aligned with the findings of the present study and corresponding targeted interventions.* |
| 7. | Approximately, 13.6% and 5.1% of young married 38 women and unmarried girls, respectively, experienced major depressive symptoms. The word approximately does not seem appropriate here as 13.6% is quite a clear number. It is not approximate. | *The word ‘*approximately’ *is removed in this revision.* |
| 8. | Please include confidence intervals for both of these figures – 13.6 and 5.1. | *13.6 (95% Confidence limit 12.2-15.2); 5.1 (95% Confidence limit 4.2-6.1)* |
| 9. | Line 57 – avoid beginning your sentences with BUT | *The sentences were rephrased.* |
| 10. | Introduction is weak and not structured well. Need to be reworked. | *Authors have modified the introduction addressing the following comments.* |
| 11. | A lot of previous work on prevalence of major depressive disorder/depression has been ignored in the introduction. Please search thoroughly and cite them in the introduction. | *The introduction of the study primarily emphasized previous research that addressed the variables utilized or aligned with in the current research. However, in response to the comment, a thorough search was conducted to incorporate additional literature concerning the prevalence of major depression, focusing on adolescents and young adults, into the revised version.* |
| 12. | After national mental health survey 2015-16, a major study by India State Level Disease Burden Initiative was published in 2020 where state wise data on 9 mental disorders, including major depressive disorders has been is presented. This was published in Lancet Psychiatry. Here is the link: <https://www.thelancet.com/journals/lanpsy/article/>PIIS2215-0366(19)30475-4/fulltext | *Authors have cited the relevant findings of this work in the introduction. Also followed this article’s discussion section in order to incorporate about implementation of findings.* |
| 13. | Theoretical framework does not need to have a heading of its own. Let it flow within the introduction. Same with hypothesis heading. | *“Theoretical framework” and “Hypothesis testing” both of these headings were removed.* |
| 14. | The multicollinearity among the independent variables was 210 measured through the variance inflation factor (VIF > 10) and its respective tolerance (<0.1). Why have you included “(VIF > 10)” in your sentence? | *A Variance Inflation Factor (VIF) value greater than or equal to 10 is a rule of thumb for severe multicollinearity. Therefore, the authors included the VIF cut-off.* |
| 15. | Please describe the nested structure and provide figures (numbers) for each level. | *The authors have given a nested model which includes the figures of both the levels (Figure 2).* |
| 16. | Line 211 Explain what is mixed-effects? Is your model a random intercept or random slope model? | *To account for the hierarchical structure of our data, we employed separate two-level mixed-effects logistic regression models for married and unmarried individuals separately. Here we have considered the random intercept model for the random effect. However, the statistical analyses section is modified to explain ‘mixed-effect” explicitly in this revision.* |
| 17. | Lines - more IPV and less IPV categories? The use of more or less does not seem appropriate. | *“More or less IPV” is changed into high and low IPV in the present revise.* |
| 18. | Line – 179: created by merging three questions – use appropriate language. You don’t merge questions to create a new variable. | *The variable on sexual assault at the community level was created by merging three items in the questionnaire. However, the sentence is rephrased in this revision.* |
| 19. | Line 242: pay attention to grammar and tenses. | *The authors have corrected the grammar and tenses of this sentence.* |
| 20. | Line 244: ICC and PCV: How are they different from each other? | *ICC reflects the proportion of variance in depression attributable to community membership, while PCV indicates the percentage reduction in unexplained variance after accounting for community-level variables compared to a null model. The authors have mentioned the definition of the terms as well as the differences within the manuscript.* |
| 21. | Odds ratio should be up to 2 digits after decimal. Three is too many. | *Authors have corrected the figures in the results section.* |
| 22. | Line 441: less odds should smaller odds. Restructure this sentence “Respondents from the communities with  more than 25% prevalence of IPV, were significantly less odds of having symptoms of major depression” – does not  make sense in its current form. | *The grammatical error in this sentence was corrected in this revision.* |
| 23. | Clarify the meaning of 'social ecology' in line 448 to enhance reader understanding | *In this study, the concept of “social ecology” was adopted from Bronfenbrenner’s Ecological Systems Theory. It was explained in the theoretical framework section of the introduction.* |
| 24. | Line 416: “The third and final model, where both individual and community-level variables were included, showed 22.3% of the unexplained variation in depression could be attributable to community and household-level factors together.” Should it be “unexplained variation” or explained variation? | *The ICC “22.3%” was a typo error. The correct figure would be 22.8 as per the table (3b). It is corrected 22.8 through the revision. ICC 22.8% in the third and final model is the proportion of the total variance that can be explained by the community level. The authors have modified the statement in this revision.* |
| 25. | Table 3b: Model fit statistics – what is (Int.)? | *Here Int. means intercept. The authors have added the full form of the used abbreviation below Table 3a and 3b.* |
| 26. | Provide explanations for ICC, LL, AIC, BIC, etc., at the bottom of the table to enhance reader comprehension. | *The concept of the mode-fit statistics used such as, ICC, PCV were explained in the methodology section. BIC was not used as an indicator of model-fit statistics. However, as recommended by the reviewer, authors have incorporated a short definition of each of these statistical term at the bottom of the table. Corresponding references are cited in the methodology section where the terms were first introduced.* |
| 27. | On what basis have you selected community level variables? Please include this in the manuscript. | *The selection of community-level variables in our multilevel model was guided by the following criteria: To begin with, Variables that could not be measured for all the participants at the individual level. Additionally, variables that reflect characteristics of the social environment likely to influence mental health outcomes for all residents within a community.*  *For example, parental violence (father beating mother) can significantly impact adolescent mental health, but this information might not be applicable to all participants due to demise of one parent or single parenthood. By* *aggregating this variable to the PSU level, we can* examine its effect of functioning at the community level. *In the revised version the justification was incorporated in the methodology section.* |
| 28. | Discussion should also include a paragraph or two focusing on the policy implications of your findings. Frame this in the context of ongoing governmental and non-governmental efforts in the country. | *In this revised version, the authors have integrated pertinent governmental and non-governmental initiatives aimed at addressing mental health concerns in India. Additionally, the discussion now elucidates how the current study findings can serve to inform and complement existing efforts, thereby contributing to the formulation and implementation of further recommendations in this domain.* |
| 29. | Line 555: “show a different set of predictors and their associations” – does not make sense – please rewrite this sentence. What do you mean by “different set” – different from??? | *The authors have modified the sentence. This clarifies that, the predictors which are associated with MDD are different for both the studied groups.* |
| 30. | Line 598: “The ongoing program on mental health issues at state level should focus on these findings and implement their intervention so that we can properly address this public health concern.” – program should focus on…findings??? It is unclear as to what authors want to convey by this sentence. Please rewrite this sentence. I would suggest you re-write the conclusion. | *In this revision the authors have revised the Conclusion of the manuscript to better articulate the implications of the study findings and align them with existing policy frameworks.* |
| 31. | Why did authors apply multilevel models? The rationale behind choosing this kind of model must be provided in the text. | *The authors decided to apply multilevel model because of two reasons. The following reasons were also incorporated in the text in the revised version of the manuscript.*  *1. The UDAYA data set had certain variables that could* *not be directly measurable at the individual participant level, yet they reflect characteristics of the social environment potentially influencing mental health outcomes for all residents within a community.*  *2. The nested structure of the UDAYA-2 data allows the generation of multilevel regression models to examine the effects of various contextual variables operating at the community level apart from determinants at the individual level* |
| Reviewer #2: |  |  |
|  | The introduction and results sections of your manuscript are well written. However, the discussion section lacks sufficient linkage to the existing literature, resulting in a gap in contextual grounding.  Create coherence in the discussion, identify the findings that conflict with other findings, and sufficiently discuss the reason for the incongruence. | *The authors have made significant revisions to the discussion section of the manuscript, aiming to enhance coherence with the theoretical framework and provide a more robust linkage to the existing literature.* |
|  | Line 444-447: Summarise your key findings | *The first paragraph of the Discussion is modified in this revision in order to summarise the key findings.* |
|  | Line 448: what do you mean by higher level. | *With refer to the concept of “social ecology” was adopted from Bronfenbrenner’s Ecological Systems Theory Higher level or higher ecological level means the level of social ecology beyond the individual level or microlevel.* |
|  | Line 448: Replace the word “enlightened” with highlighted/delineated. | *The word is replaced in this revision.* |
|  | Line 451: cite the evidence of "degraded values and regressive community" from your study or else this is blanket  statement. | *By using this phrase "degraded values and regressive community" the authors tried to describe the social disorganization especially for the women in community owing to higher sexual assaults and dowry-related humiliation. With higher social disorganization participants are more likely to be intimidated in terms of being socially condemned or humiliated. In this context the concerned phrase was used as a supportive statement rather a blanket statement.* |
|  | Line 454: are you reporting lower depression in the instance of male perpetuated violence against women in the family?? | *In this context, the authors explained that in the communities where male perpetuated family violence (in the form of father beaten mother) was reported by the unmarried girls, participants less likely to experience depression.* |
|  | Line 453-455: ? | *The authors posit that the high prevalence of social condemnation, domestic violence, and sexual abuse within these communities suggests a broader social ecological context characterized by weakened social norms. This environment may create a sense of intimidation and vulnerability among the participants, potentially contributing to their susceptibility to depression. In this context the phrase “degraded values and a regressive community” was used as a supportive statement rather a blanket statement.* |
|  | Line 478: Does it prevent mental health? | *Existing research suggests that higher prevalence of being a minority can be linked to higher depression rates attributed to intercultural conflict, neighbourhood discrimination, and unfair treatment. The authors tried to explain that in the present study, the lower odds of MDD among the young married OBC women might be due to a sense of community within the dominant social group in the region.* |
|  | Line 480-489: delineate the relevant findings, add context and implication of findings with respect to variables related to education | *Our analysis revealed a counterintuitive finding: higher education was associated with increased odds of MDD. Furthermore, a significant association was found between MDD risk and both employment status (unemployed or looking for jobs) and paid employment. This suggests that educational attainment may lead to higher aspirations, and the unfulfilled aspirations associated with unemployment or an unsatisfactory job could contribute to the burden of MDD among the study participants. We have rephrased the text in the manuscript to explicitly convey this complex interplay between education, employment, and mental health.* |
|  | Line 501-511: incoherent sentences. | *In this segment the authors provided the potential justification of the association of MDD with age at marriage, hours of physical activity, hours of screentime (in terms of content watching and use of social media). Our analyses had shown an unconventional finding. The odds of MDD were lower with higher age at marriage, lower hours of physical exercise and higher hours of screentime. Prior sentences are modified by the authors.* |
|  | Line 510: if so, contextualise with studies that suggest association between increase in screen time and mental health. | *While previous research suggests an inverse association between screen time and mental health problems, with higher screen time linked to increased risk, our study observed a contrasting trend. One potential explanation could be the nature of the content participants were consuming. Studies like Benton (2022) highlight the potential mood-boosting effects of watching movies.* *These findings suggest to investigate the screen time content, not merely duration, is a more nuanced influence on mental health. This revised manuscript contains more explicit justification on the abovementioned account.* |
